# Immunological findings in psychotic syndromes: a tertiary care hospital's CSF sample of 180 patients

**DOI:** 10.3389/fnhum.2015.00476

**Published:** 2015-09-10

**Authors:** Dominique Endres, Evgeniy Perlov, Annette Baumgartner, Tilman Hottenrott, Rick Dersch, Oliver Stich, Ludger Tebartz van Elst

**Affiliations:** ^1^Section for Experimental Neuropsychiatry, Department for Psychiatry and Psychotherapy, University Medical Center FreiburgFreiburg, Germany; ^2^Department for Neurology, University Medical Center FreiburgFreiburg, Germany

**Keywords:** CSF, psychotic syndrome, schizophrenia, antineuronal autoantibodies, immunological encephalopathy

## Abstract

Immunological mechanisms and therapy approaches in psychotic syndromes were recently supported by the discovery of autoantibody-associated limbic and non-limbic encephalitis. However, how clinical diagnostic procedures in psychiatry should be adapted to these new insights is still unclear. In this study, we analyzed the cerebrospinal fluid (CSF) and neuroimmunological alterations and their association with cerebral MRI (cMRI) and electroencephalographic (EEG) findings. From 2006 to 2013, we acquired 180 CSF samples from psychotic patients. Between 2006 and 2009, CSF examinations were only performed in cases in which organic brain disease was suspected. Since then, this procedure has been integrated into our routine diagnostic workup. CSF basic diagnostics were supplemented by measuring antineuronal antibodies against intracellular synaptic antigens, antibodies against intracellular onconeural antigens, antibodies against neuronal cell surface antigens and thyroid antibodies. In addition, cMRIs and EEGs were conducted. We found white cell counts elevated in 3.4% of the cases, albumin quotient elevated in 21.8%, and protein concentration elevated in 42.2%. Evidence of intrathecal immunoglobulin synthesis was found in 7.2% of the cases. Antibodies measured against neuronal cell surface antigens were positive in 3.2%. Reactivity on antibodies against intracellular onconeural antigens were detected in 3.5%. Serum thyroid antibodies were elevated in 24.7%. Abnormalities were found in 39.5% of cMRIs and in 34.3% of EEGs. The main finding of our study was the high prevalence of CSF and autoantibody abnormalities in 54.4% of psychotic patients. In combination with cMRIs and EEGs, 75.6% showed abnormal findings. Our results are discussed with regard to the concept of immunological encephalopathy. Future studies should analyze the efficacy of immunomodulatory therapies.

## Introduction

Psychosis was identified as a possible immunological disease as early as 1930 in the work of Lehmann-Facius among others. He described a highly specific method called “brain–lipoid reaction” to detect immunological signals in psychotic patients. This in turn led to the autoimmune hypothesis of schizophrenia (Roeder, 1939). Immunological concepts were recently supported by autoantibody-associated limbic and non-limbic encephalitis dissembling psychotic syndromes. In this context, antineuronal antibodies against intracellular synaptic antigens, intracellular onconeural antigens, and neuronal cell surface antigens could be distinguished. Neuronal cell surface antigens appear to be particularly associated with psychotic symptoms (Prüss et al., 2010; Dalmau et al., 2011; Vincent et al., 2011). Moreover, steroid responsive encephalopathy associated with autoimmune thyroiditis (SREAT) has received an increased level of interest in recent years because it was identified as the cause for classical psychotic and affective disorders in several cases (Castillo et al., 2006).

### Role of cerebrospinal fluid (CSF) analysis in clinical diagnostics of psychotic patients

Cerebrospinal fluid (CSF) investigation is the most precise method of detecting and characterizing central nervous system inflammatory processes, even when cerebral magnetic resonance imaging (cMRI) or electroencephalographic (EEG) results are normal. In most guidelines (e.g., Nice guidelines, http://www.nice.org.uk; German S3-Praxisleitlinie, www.dgppn.de), CSF examinations are not recommended in the diagnostic routine work up of psychotic or affective disorders. However, there have been several recent reports pointing to good response to immune therapy in patients with classical psychiatric manifestations of psychotic or affective disorder without organic symptoms or signs (van Elst et al., 2011; Chang et al., 2013). These observations put the psychiatric clinician in a difficult position because it is hard to judge when to initiate and when to leave CSF analyses in an individual case.

### Rationale of our study

At the university clinic of psychiatry and psychotherapy Freiburg, traditionally CSF studies have always been done in case of psychotic patients if there were any signs pointing to possible neuroinflammatory features, such as atypical and sudden-onsets, seizures, or suspicious cMRI/EEG findings. Following own experiences with patients with limbic encephalitis presenting like schizophreniform syndromes, since June 2009, we offered CSF analyses as part of our routine work up to all psychotic patients who were admitted for diagnostic purposes. With these data at hand, the aim of this retrospective study was threefold. (1) We wanted to investigate the frequency of CSF and neuroimmunological alterations and their association with cMRI and EEG findings in psychotic syndromes. We hypothesized to find increased prevalences of CSF-basic laboratory abnormalities and autoantibody rates in psychotic patients as well as an association between CSF and cMRI/EEG abnormalities. (2) We wanted to analyze how a change in diagnostic approach might affect the respective immunological findings. Could it be that our new diagnostic approach to offer CSF studies to all psychotic subjects is excessive and produces lots of negative findings? (3) We wanted to analyze on a case by case level, whether the findings of immunological signals have therapeutic implications for our patients.

## Participants and methods

The study received approval from the local ethics committee of the University of Freiburg (EK-Fr 609/14). All patients included gave written informed consent for CSF studies during the clinical diagnostic work up.

### CSF collective

From 2006 to October 2013, we acquired 180 CSF samples from psychotic patients at the Clinic of Psychiatry and Psychotherapy of Freiburg University Hospital. Lumbar punctures (LPs) were generally performed during the initial manifestation of psychotic symptoms. Three clinically defined subgroups were analyzed: (1) patients with a schizophreniform syndrome, (2) patients with a schizoaffective syndrome, and (3) patients with psychotic syndromes in the context of other disorders (Table 1).

**Table 1:** **Subgroup classification and available number of data sets**.

**Psychotic CSF-collective**	**Number of patients**
**(1) Schizophreniform syndrome group**	**132**
Schizophrenia	104
Delusional disorder	4
Acute polymorphic psychotic disorder	18
Schizotypal disorder	2
Substance-induced psychosis	4
**(2) Schizoaffective syndrome group**	**43**
**(3) Psychotic syndromes in the context of other disorders**	5^*^
**Diagnostic measurements**	**Number of samples**
**CSF basic diagnostics** (white blood cell count, protein concentration, albumin quotient, and intrathecal immunoglobulin synthesis)	180
**Intracellular synaptic antigens** (GAD, amphiphysin) and **intracellular onconeural antigens** (Yo, Hu, Ri, Cv2/CRMP5, Ma1, Ma2, SOX1)	142
**Antibodies against neuronal cell surface antigens** (NMDAR, AMPA-1/2-R, GABA-B-R, VGKC-complex)	96 (*Freiburg*) + 29^**^(Weatherall Institute of Molecular Medicine at the John Radcliffe Hospital [Oxford, United Kingdom])
**Thyroid-antibodies**	
Anti-thyroid peroxidase antibodies	73
Anti-thyroglobulin antibodies	70
Thyroid-stimulating hormone receptor antibodies	33
**Electroencephalography data sets**	175
**Magnetic resonance imaging data sets**	152

* Psychotic syndrome group in the context of other disorders is consisting of (1) Morbus Wilson, (2) presumed viral encephalitis, (3) prodromal symptoms of derealization and depersonalization, (4) prodromal symptoms within the framework of autistic spectrum disorder, and (5) acute state of confusion with psychotic symptoms.

** *Only NMDAR and VGKC-complex antibodies. GAD, glutamic acid decarboxylase; Yo/ Hu/ Ri, abbreviations of first patients' name; Cv2/CRMP5, anti-collapsin response-mediator protein; Ma1/ Ma2, 37 and 40 kDa neuronal proteins; SOX1, sry-like high-mobility group box 1; NMDAR, N-methyl-D-aspartat-receptor; AMPA-1/2-R, α-Amino-3-hydroxy-5-methyl-4-isoxazole-propionic acid receptor; GABA-B-R, γ-aminobutyric acid receptor; VGKC-complex, voltage gated potassium channels complex*.

### Measurement protocols

From 2006 to June 2009, we obtained 34 CSF analyses following the traditional approach i.e., LP was done only if there were specific symptoms and signs pointing to an organic cause of psychotic symptoms. This corresponds to 9.7 LPs per year. Since June 2009, we have introduced LP as a standard procedure in all psychotic patients with acute de novo psychotic symptoms under our care. We measured 146 cases until October 2013 (34.4 LPs per year).

### Immunological assessment

In all cases, paired CSF and serum samples were taken at the same time. CSF diagnostics were performed in the CSF laboratory of the Department of Neurology. CSF white blood count (WBC) and cytological differentiation of CSF sediment were established with manual microscopy (Leica DMRB, Germany) using a Fuchs-Rosenthal counting chamber (Hecht-Assistant, Germany). Basic quantitative protein diagnostics included total CSF protein concentration, albumin, and Ig G, M, and A concentrations in CSF and serum, respectively (ProSpect System, Siemens, Erlangen, Germany). For detection of a blood–brain barrier (BBB) dysfunction, we calculated the age-related albumin quotient since this marker is accepted as the “gold standard” for the estimation of the integrity of the BBB (Reiber and Peter, 2001). For determination of oligoclonal bands (OCBs), we used isoelectric focusing followed by immunofixation (Hydragel Isofocusing, Sebia, France). Intrathecal Ig synthesis was considered significant if intrathecal Ig fraction-values exceeded 10% in the “Reibergram”-analysis (Reiber and Peter, 2001) and/or if OCBs were present exclusively or predominantly in CSF according to the criteria of the European experts' consensus (Andersson et al., 1994). A commercially available immunoblot employing recombinant neuronal antigens as the substrate (Yo, Hu, Ri, Cv2/CRMP5, Ma1, Ma2, SOX1, amphiphysin, and GAD) was used for qualitative detection of antineuronal antibodies against onconeural intracellular or synaptic antigens (ravo Diagnostika, Freiburg, Germany), as has been the case since 2006. CSF antibodies against neuronal cell surface antigens (NMDAR, AMPA-1/2-R, GABA-B-R, and VGKC-complex [LGI1, Caspr2]) were detected using a monospecific cell-based (transfected HEK cells) indirect immunofluorescence assay (Euroimmun, Luebeck, Germany), as has been the case since 2011. Prior to this, samples were sent to the reference laboratory at the Weatherall Institute of Molecular Medicine at the John Radcliffe Hospital (Oxford, United Kingdom) for measuring the serum anti-voltage gated potassium channel (VGKC) complex and anti-N-methyl-D-aspartat-receptor (NMDAR) antibodies. Serum thyroid autoantibodies, including anti-thyroid peroxidase, anti-thyroglobulin, and thyroid-stimulating hormone receptor antibodies, were assessed only in cases of clinical suspicion of thyroid disorder (i.e., abnormalities in thyroid hormones) using electrochemiluminescence immunoassay tests (Roche, Basel, Switzerland). The cMRI and EEG datasets were obtained from clinical records; EEGs were analyzed visually by in-house physicians, while cMRI datasets were analyzed by experienced senior neuroradiologists. Table 1 provides an overview of the number of datasets.

### Data handling and statistical analysis

All laboratory data, clinical information and cMRI and EEG datasets were carefully researched and entered into a data bank (SPSS 20, Statistical Package for the Social Sciences). Mainly, descriptive statistics were calculated for the psychotic patient group. For group comparison (respective age) between patients with and without clear-cut antibody abnormalities, we used a Mann–Whitney *U*-test. Moreover, we performed Pearson correlation for correlation analyses between age and CSF markers.

## Results

### Demographic data

Of the 180 examined psychotic patients, 101 were female and 79 were male; the mean age was 34.67 (standard deviation ±14.7) years.

### CSF basic diagnostics and immunological findings

Table 2A summarizes the basic CSF findings. Overall, 3.4% of our psychotic patients displayed increased WBC counts. Seventy-six of the 180 patients or 42.2 % showed an increased protein concentration, 39 patients or 21.8% showed an increased albumin quotient and in 13 patients or 7.2% there were OCBs. Age significantly correlated with the protein concentration (*r* = 0.185, *p* = 0.013, *N* = 180) and the albumin quotient (*r* = 0.250, *p* = 0.001, *N* = 179). There was no significant correlation between age and WBC (*r* = −0.068, *p* = 0.368, *N* = 179) or age and IgG-index (*r* = −0.047, *p* = 0.534, *N* = 180). Table 2B illustrates the specific findings with respect to antineuronal antibodies. In 4 cases or 3.2% we found antibodies against neuronal cell surface antigens. Antibodies against intracellular onconeural antigens were found in 5 cases or 3.5%. Thyroid-stimulating hormone receptor antibodies were increased in 3.1% of investigated cases, thyroid peroxidase antibodies in 17.8% and thyroglobulin antibodies in 15.7%.

**Table 2:** **(A) Basic CSF findings and (B) Autoantibody results in entire psychotic patient group and associated neuropsychiatric syndromes (http://www.dgn.org/leitlinien/inhalte-nach-kapiteln)**.

**(A) Basic CSF findings**	**Measurement**	**Number of cases**	**Frequency of abnormalities**	**Threshold**	
CSF basic diagnostics (*N* = 180)	White blood cell count^*^	1–4 cells: 173 >5 cells: 6 (cell counts: 6, 11, 15, 59, 72, 101)	3.4%	*< 5/μl*	
	Protein concentration	↔: 104 ↑: 76	42.2%	*< 450 mg/l*	
	Albumin quotient^**^	↔: 140 ↑: 39	21.8%	*< 40 years: 6.5 × 10^−3^* *40–60 years: 8.0 × 10^−3^* *>60 years: 9.3 × 10^−3^* (Stich et al., 2013)	
	Intrathecal immunoglobulin-synthesis	No: 167 Yes: 13 - OCB restricted to CSF ^***^: 10 - OCB mirror pattern ^***^: 3	7.2%; OCB restricted to CSF: 5,6%; OCB mirror pattern: 1.7%	*Immunoglobulin-G-Index ≤ 0,7 mg/l*, *No immunoglobulin-G/-A/-M synthesis rate*, *No OCBs*	
**(B) Antibody findings**	**Antibodies**	**Number of cases**	**Relative frequency**	**Explanation**	**Neuropsychiatric syndromes**
Antibodies against neuronal cell surface antigens (*N* = 125)	Anti-VGKC-complex-antibodies	3 cases (of 125)	2.4%	*Antibodies against voltage gated potassium channels*	Limbic encephalitis, Morvan syndrome
	Anti-NMDAR-antibodies	1 case (of 125)	0.8%	*Antibodies against N-methyl-D-aspartat-receptor*	Encephalopathy, epilepsy, dementia, psychosis
	Anti-AMPAR-antibodies	0 cases (of 96)	0%	*Antibodies against α-Amino-3-hydroxy-5-methyl-4-isoxazole-propionic acid receptor*	Limbic encephalitis, atypical psychosis, epilepsy
	Anti-GABA-B-antibodies	0 cases (of 96)	0%	*Antibodies against γ-Aminobutyric acid*	Limbic encephalitis, epilepsy
	Anti-mGluR1/mGluR5-antibodies	Not measured	-	*Antibodies against metabotropic glutamate receptors*	mGluR1: Cerebellar ataxia mGluR5: Limbic encephalitis, Ophelia-syndrome
Antibodies against intracellular synaptic antigens (*N* = 142)	Anti-GAD-antibodies	No cases	0%	*Antibodies against glutamic acid decarboxylase*	Stiff-person-syndrome, limbic encephalitis, epilepsy
	Anti-amphiphysin-antibodies	No cases	0%		Stiff-person-syndrome, limbic encephalitis, cerebellar degeneration
Antibodies against intracellular onconeural antigens^****^ (*N* = 142)	Anti-Yo-reactivity	3 cases	2.1%	*Abbreviation of first patients name*	Subacute cerebellar degeneration
	Anti-Hu-reactivity	1 case (low positive)	0.7%	*Abbreviation of first patients name*	Limbic encephalitis, cerebellar degeneration, epilepsy
	Anti-cv2 (CRMP5)-reactivity	1 case (low positive)	0.7%	*CRMP: Anti-collapsin response-mediator protein*	Limbic encephalitis, cerebellar degeneration
	Anti-Ri-reactivity	No cases	0%	*Abbreviation of first patients name*	Limbic encephalitis, cerebellar degeneration
	Anti-Ma1/-Ma2-reactivity	No cases	0%	*37 and 40 kDa neuronal proteins*	Limbic encephalitis, cerebellar degeneration
	Anti-SOX1-reactivity	No cases	0%	*Sry-like high-mobility group box 1*	Limbic encephalitis, cerebellar degeneration
	**Antibodies**	**Mean ± SD**	**Relative frequency**	**Threshold**	**Neuropsychiatric syndromes**
Serum thyroid antibodies	Thyroid-stimulating hormone -receptor antibodies (*N* = 33)	↔: 32 ↑: 1 Mean ± SD: 0,58 ± 0,51 IU/l	3.1%	< 1.75 IU/l	Graves' disease
	Thyroid peroxidase antibodies (*N* = 73)	↔: 60 ↑: 13 Mean ± SD: 53.0 ± 161.8 IU/l	17.8%	< 34 IU/l	SREAT
	Thyroglobulin antibodies (*N* = 70)	↔: 59 ↑: 11 Mean ± SD: 73,8 ± 157,0 IU/l	15.7%	< 115 IU/l	SREAT

* White blood cell count was measured in 179 of 180 samples;

** Albumin quotient was measured in 179 of 180 samples;

*** Oligoclonal bands restricted to CSF were found exclusively or predominant in CSF, while a oligoclonal band mirror pattern shows identical oligoclonal bands in CSF and serum;

**** *High concentrations are associated with paraneoplastic neurological syndromes. SD, standard deviation; ↔, not over threshold; ↑, increased over standard value; OCBs, Oligoclonal bands; IU/l, International Units per liter; SREAT, Steroid-responsive encephalopathy associated with autoimmune thyroiditis*.

Table 3 specifies all clinical relevant findings of the antibody positive patients. Three of the nine patients with antineural antibodies displayed clear cut additional neurological symptoms (epileptic seizures) in addition to the schizophreniform or schizoaffective syndrome earlier or later in the course of the disease. This was not the case in the other 6 patients. The EEG was abnormal in 4 of 9 patients. The cMRI was completely normal in 4 of 9 cases. However, the abnormalities in the remaining 5 of 9 were unspecific with focal atrophy being the most common finding. An FDG-PET was done in 4 of 9 patients with mixed results.

**Table 3:** **Findings in antibody-positive patients**.

**Antibody**	**Psychiatric syndrome**	**Neurological abnormalities**	**EEG**	**cMRI**	**FDG-PET**	CSF^*^
**ANTIBODIES AGAINST NEURONAL CELL SURFACE ANTIGENS**						
Low titre VGKC-antibodies (49 years, female)	Schizoaffective syndrome	Changing consciousness	Normal	Normal	Not performed	Protein concentration 474 mg/l
Low titre VGKC-antibodies (20 years, female)	Schizophreniform syndrome	Normal	Intermittent generalized slow activity	Normal	Not performed	Protein concentration 604 mg/l, albumin quotient 8.7
Low titre VGKC-antibodies (25 years, male)	Schizophreniform syndrome	Stupor, aphasia, seizures	Continuous generalized slow activity, intermittent bitemporal spikes	A singular right frontal white matter lesion	Not performed	WBC count 72/μl, protein concentration 470 mg/l, OCBs restricted to CSF
Anti-NMDAR-antibodies (30 years, female)	(Catatonic) Schizophreniform syndrome	Seizures	Intermittent regional slow activity	Perisylvic/temporal accentuated atrophy	Global cortical hypometabolism, more pronounced on left side	Protein concentration 561 mg/l, albumin quotient 8.7
**ANTIBODIES AGAINST INTRACELLULAR ANTIGENS**						
Anti-Yo-reactivity (23 years, female)	Schizophreniform syndrome	None	Normal	Normal	Unspecific strong metabolism striatal and in bone-marrow	Normal
Weak anti-Hu-reactivity (24 years, male)	Schizophreniform syndrome	None	Intermittent generalized slow activity and sporadic epileptic pattern	Normal	Normal	Protein concentration 1510 mg/l, albumin quotient 20.8
Weak anti-cv2/CRMP5-reactivity (50 years, male)	(Catatonic) Schizophreniform syndrome	Parkinsonism, seizures	Normal	Parietal accentuated generalized atrophy, frontal white matter lesions	Not performed	Protein concentration 472 mg/l, albumin quotient 10.6
Anti-Yo-reactivity (20 years, female)	Schizoaffective syndrome	Mild dysdiadochokinesia	Normal	Fronto-parieto-cerebellar atrophy	Moderate hypometabolism of cerebellar hemispheres	Normal
Weak anti-Yo-reactivity (18 years, male)	Schizophreniform syndrome	None	Normal	Mega-cisterna magna, enlarged cella media right, enlarged temporal horn right, sporadic minor hemorrhaging parietal after head injury	Not performed	Normal

* *Only CSF abnormalities are mentioned. cMRI, cerebral MRI; FDG-PET, fluorodeoxyglucose positron emission tomography; VGKC-complex, voltage gated potassium channels complex; NMDAR, N-methyl-D-aspartat-receptor; Yo/Hu, abbreviations of first patients's name; Cv2/CRMP5, anti-collapsin response-mediator protein*.

Patients with clear-cut antibody findings (*n* = 9) were younger on average compared with antibody negative patients (*n* = 171), however, these differences were not significant (28.78 ± 12.26 vs. 34.98 ± 14.77; *U* = 993.5, *p* = 0.142).

### Effect of diagnostic protocol on findings

To analyze how the introduction of a new diagnostic protocol may affect respective results, we compared the cohort obtained between 2006 and June 2009 to the one obtained from data since 2009. Table 4 summarizes respective findings. It illustrates that the introduction of routine CSF studies in 2009 led to a lower detection rate of increased WBC counts from 9.1 to 2.1%. In contrast, the high detection rate of protein abnormalities (increased protein concentration: 47.1% down to 41.1%; albumin quotient: 23.5% down to 21.4%) and intrathecal immunoglobulin synthesis (8.8% down to 6.8%) was not altered in a relevant way and was very high even in routine assessment of CSF in psychotic patients.

**Table 4:** **CSF-basic diagnostics sorted by date (2006–2009: LP in suspicious cases; 2009–2013: LP as a standard screening procedure) and patient subgroups**.

	**Date**	**Threshold**	**Schizophreniform syndrome (*N* = 132)**	**Schizoaffective syndrome (*N* = 43)**	**Psychotic syndromes in the context of other disorders (*N* = 5)**	**Whole group of psychotic syndrome (*N* = 180)**
White blood cell count^*^	2006–2009	< 5 cells ≥5 cells	24/25 (96%) 1/25 (4%)	6/7 (85.7%) 1/7 (14.3%)	0/1 (0%) 1/1 (100%)	30/33 (90.9%) 3/33 (9.1%)
	2009–2013	< 5 cells ≥5 cells	105/107 (98.1%) 2/107 (1.9%)	34/35 (97.1%) 1/35 (2.9%)	4/4 (100%) 0/4 (0%)	143/146 (97.9%) 3/146 (2.1%)
Protein concentration	2006–2009	< 450 mg/l ≥450 mg/l	14/25 (56%) 11/25 (44%)	4/8 (50%) 4/8 (50%)	0/1 (0%) 1/1 (100%)	18/34 (52.9%) 16/34 (47.1%)
	2009–2013	< 450 mg/l ≥450 mg/l	63/107 (58.9%) 44/107 (41.1%)	20/35 (57.1%) 15/35 (42.9%)	3/4 (75%) 1/4 (25%)	86/146 (58.9%) 60/146 (41.1%)
Albumin quotient^*^	2006–2009	Age-dependent: Increased:	19/25 (76%) 6/25 (24%)	6/8 (75%) 2/8 (25%)	1/1 (100%) 0/1 (0%)	26/34 (76.5%) 8/34 (23.5%)
	2009–2013	Age dependent: Increased:	83/106 (78.3%) 23/106 (21.7%)	28/35 (80%) 7/35 (20%)	3/4 (75%) 1/4 (25%)	114/145 (78.6%) 31/145 (21.4%)
Intrathecal immuno-globulin-synthesis	2006–2009	No: Yes:	24/25 (96%) 1/25 (4%)	7/8 (87.5%) 1/8 (12.5%)	0/1 (0%) 1/1 (100%)	31/34 (91.2%) 3/34 (8.8%)
	2009–2013	No: Yes:	100/107 (93.5%) 7/107 (6.5%)	32/35 (91.4%) 3/35 (8.6%)	4/4 (100%) 0/4 (0%)	136/146 (93.2%) 10/146 (6.8%)

* *Measured only in 179 of 180 samples*.

### cMRI and EEG

cMRI abnormalities were detected in 39.5% of the cases (60 of 152). Of the available 175 EEG datasets, 60 showed pathological abnormalities (34.3%). Intermittent generalized slow activity was found most frequently (18.3%; Table 5).

**Table 5:** **Cerebral Magnetic Resonance Imaging (cMRI) and Electroencephalography (EEG) Abnormalities**.

Localization of cMRI abnormalities^*^ (*N* = 152)	**Frequency (%)**
White matter	26 (17.1%)
Cortical atrophy	11 (7.2%)
Temporal lobe	4 (2.6%)
Frontal lobe	3 (2.0%)
Parietal/ occipital lobe	2 (1.3%)
Cerebellum	1 (0.7%)
Brainstem	1 (0.7%)
Brain's ventricle abnormalities	5 (3.3%)
Thalamus	3 (2.0%)
Unspecific changes	4 (2.6%)
cMRI abnormalities (absolute)	60 (39.5%)
EEG abnormalities^*^ (*N* = 175)	**Frequency (%)**
Continuous generalized slow activity	7 (4%)
Continuous regional slow activity	0 (0%)
Intermittent generalized slow activity	32 (18.3%)
Intermittent regional slow activity	14 (8%)
Epileptic pattern	7 (4%)
EEG abnormalities (total)	60 (34.3%)

* *Only the predominant cMRI lesion or EEG abnormality is listed for each patient. cMRI, cerebral MRI*.

### Overall abnormalities

CSF and serum analyses (including antineuronal and anti-thyroid antibodies) showed abnormalities in 98 of the 180 cases (54.4%). cMRI or EEG abnormalities were found in 91 of the 180 psychotic patients (50.6%). Total abnormalities in CSF analysis and cMRI/EEG analysis were detected in 136 of the 180 cases (75.6%).

## Discussion

The main finding of our study was the high percentage of CSF and autoantibody abnormalities in 54.4% of all psychotic patients and overall organic abnormalities (including cMRI and EEG) in 75.6%. Before discussing the possible relevance of these findings, we have to stress the shortcomings of our open study.

### Limitations

The entire study is open and followed clinical practice. Therefore, while the ecological validity might be high, the sample was generated in a very unsystematic way. The total collective includes a group of patients with suspected organic brain disease identified between 2006 and 2009 and a more systematic screening group since 2009. This includes all psychotic patients who gave their written consent for LP. While the majority of patients agreed to all proposed diagnostic procedures could not clarify retrospectively how many patients rejected CSF studies. Therefore, this sample cannot be regarded as representative. Still, since this issue is rather new to psychiatry, it is difficult to obtain data from more systematic samples. To our knowledge, this is the first study published in the literature looking at a large clinical sample of unselected consecutively diagnosed psychotic patients who were comprehensively investigated using CSF analysis, the measurement of different neuroimmunological markers (autoantibodies), EEG and cMRI. The major shortcoming of this study is the fact that we did not analyze a control group for comparison. However, this is an open clinical observational study, and for ethical reasons, we would not have obtained approval for doing LPs in healthy controls from our ethics committee, due to possible complications. The high rates of organic pathologies might also be influenced by medication effects, comorbidities, or patient history (e.g., EEG findings are very sensitive for such influencing factors). In spite of these limitations, we feel that this study is of clinical significance because we were able to collect a large sample of psychotic patients, who were diagnosed by experienced senior psychiatrists in a tertiary care hospital on a strictly clinical basis. Presently CSF studies do not belong to the recommended diagnostic procedures. However, given the good therapeutic response to immunomodulatory therapies in some patients with immunological encephalopathy the issue of whether these recommendations should be modified and adapted to the latest findings in neuroimmunology is of obvious clinical relevance.

### The effect of more elaborated diagnostic procedures

One might have hypothesized that the change in diagnostic protocol, essentially introducing CSF examination as a routine diagnostic measure, would result in a majority of negative findings. This, however, was not the case. While the prevalence of elevated CSF WBC count went down from 9.1 to 2.1%, the same was not true for all other global CSF basic signals. In particular, we found strong evidence pointing to disturbed BBB function in psychotic patients.

### Role of CSF basic diagnostics in psychotic syndromes

Several studies analyzing CSF in schizophrenia have reported differences, especially in BBB function. Most previous studies are older, and therefore, were assessed under different environmental and technical frameworks (Supplemental Table 1).

#### WBC count

In earlier studies, WBC count showed no abnormalities overall in psychotic patients (Vasic et al., 2012). However, in acutely psychotic patients, there were reports of higher rates of activated lymphoid cells and mononuclear phagocytes, which might be indicative of microglial activation, which—according to some reports—normalizes over the course of the disease (Nikkilä et al., 1999, 2001). In our sample, we found elevated WBC counts in 3.4%, which might be evidence of discrete acute autoimmune or infectious inflammatory processes in a small subgroup of psychotic patients.

#### BBB

Increased BBB permeability with elevated protein concentration and CSF-to-serum albumin quotients have been described in previous studies (Vasic et al., 2012). Bechter et al. for example, reported a moderate BBB dysfunction in 29% of treatment-resistant affective and schizophrenic spectrum disorder patients (Bechter et al., 2010). In particular, albumin, which is produced only in the liver, is accepted to be the “gold standard” for detecting BBB permeability by measuring the serum/CSF quotient (Reiber and Peter, 2001; Vasic et al., 2012). Following older neurophysiological models, a high albumin quotient was interpreted as a “leakage” of BBB. Recent concepts have also discussed a reduced CSF flow caused by low CSF production rate, increased flow resistance in the subarachnoid space, or reduced outflow into venous blood via the arachnoid villi as further causal mechanisms (Reiber, 1994). In our study, we found increased protein concentrations in 42.2% of our patients and an elevated albumin quotient in 21.8%. While several case studies illustrate that immunomodulatory therapy may produce very positive results in single cases, it is not yet clear whether evidence for BBB dysfunction should trigger therapy courses (e.g., with steroids). Future research will have to answer this question.

#### Intrathecal immunoglobulin synthesis

We found an intrathecal humoral immune response, indicating chronic CSF inflammation, in 7.2% of our patients. Due to the chronic and relapsing character of schizophrenia, which is similar to multiple sclerosis, a chronic low-level central nervous system inflammation in a subgroup of psychotic patients was postulated by some authors (Bechter et al., 2010). For example, Bechter postulated the mild encephalitis hypothesis for schizophrenia at least for subgroups of schizophreniform patients (Bechter, 2013). Findings of intrathecal immunoglobulin synthesis may support such notions at least for a small subgroup of psychotic patients.

### Antibodies against neuronal cell surface antigens

Antibodies against neuronal cell surface antigens are particularly associated with psychotic syndromes. Table 2 includes an overview of established autoantibodies and associated neuropsychiatric syndromes. Anti-N-methyl-D-aspartat-receptor (NMDA-R) encephalitis for example begins mainly with an unspecific prodromal period, which is characterized by headache and fever and followed by a psychiatric period of anxiety, paranoia, delusions, short-term memory loss, and disintegration of language to the point of mutism (Prüss et al., 2010; Dalmau et al., 2011; Vincent et al., 2011). Therefore, anti-NMDAR encephalitis is an important differential diagnosis for catatonic schizophrenia. In the course of the disease, patients may suffer from hypoventilation, seizures, and autonomic instability (Prüss et al., 2010; Dalmau et al., 2011). The detection of anti-NMDAR antibodies is clinically important due to the high efficacy of immunomodulatory therapy options, including intravenous corticosteroids, plasma exchange, intravenous immunoglobulins, azathioprine, or monoclonal antibodies (e.g., rituximab) (Peery et al., 2012). In our collective, we found anti-NMDAR-IgG antibody positivity in serum in only one case. In a retrospective study by Steiner et al. a far higher number (9.9%) of low-titer anti-NMDA receptor antibodies were found in schizophrenic patients (Steiner et al., 2013). A recent study by Hammer et al. again showed a high prevalence of these antibodies in schizophrenic patients (8.6%) but, interestingly, an even higher prevalence in healthy controls (10.8%) (Hammer et al., 2014). Steiner and colleagues have in the meantime re-analyzed their sample, now also finding an equal percentage of anti-NMDAR antibodies in healthy controls (Steiner et al., 2014). However, in our clinic in Freiburg, we measured only IgG antibodies, Steiner et al. and Hammer et al. measured also IgA and IgM antibodies (Steiner et al., 2013; Hammer et al., 2014). Looking only on IgG-NMDR-antibodies, we found similar prevalence rates to those of previous studies. In the studies by Steiner et al. and Hammer et al. only the sera of psychotic patients and controls were analyzed (Steiner et al., 2013; Hammer et al., 2014), in our study, we mostly analyzed CSF samples. Looking at the high prevalence rates of NMDAR antibodies in healthy subjects, it may be assumed that positive antibodies in the serum probably must be associated with another mechanism, such as disturbed BBB dysfunction, in NMDAR-antibody carriers to enter a state of immunological encephalopathy (Hammer et al., 2014). Therefore, serum measurement and CSF antibody status, combined with CSF basic diagnostics, might be useful to detect such constellations.

We also found anti-VGKC complex antibodies in 2.4% of our collective (three cases). Anti-VGKC complex encephalitis often leads to limbic encephalopathy and symptoms of seizures, memory loss, and affective symptoms (Reid et al., 2009; Radja and Cavanna, 2013). However, there are cases were such patients at least initially presented with classical psychiatric syndromes, such as acute schizophreniform disorder (van Elst et al., 2011; Gnanavel, 2014).

### Antineuronal antibodies against intracellular onconeural antigens

Interestingly, we also found antineuronal antibodies against intracellular onconeural antigens in 3.5% of our patients (Tables 2B, 3; five cases). In particular, anti-Yo reactivity was detected in three patients (2.1%). Anti-Yo antibodies target Purkinje cells of the cerebellum and are the most common reason for paraneoplastic cerebellar degeneration (Hasadsri et al., 2013; Stich and Rauer, 2014). However, none of our patients had cancer. A recent study by Laadhar et al. showed an association between psychiatric diseases and antibodies against intracellular neuronal antigens. They described confirmed, well-characterized antineuronal antibodies (reproducible in a confirmation test) in five (anti-Yo in three, anti-Ri in two) of 143 patients, and none in the control group (Laadhar et al., 2015). Another large study found anti-Yo antibodies in 0.4% of 1378 schizophrenic patients, but also in 0.6% of healthy controls (Dahm et al., 2014). It was previously assumed that these well-characterized antineuronal antibodies are mostly associated with paraneoplastic neurological syndromes. Low-titre antibodies without neurological symptoms are also known in cancer patients. Our results in combination with the data from Laadhar et al. and Dahm et al. provide the first evidence of the existence of low-titre onconeural antibodies in psychiatric patients (Dahm et al., 2014; Laadhar et al., 2015). Otherwise, high percentages in healthy controls raise the question about the pathophysiological meaning of such findings. In any case, positive antibody findings should result in organic clarification. We recently published the case of a 20-year-old female patient with low-titre anti-Yo positivity with intrathecal synthesis and fronto-parieto-cerebellar atrophy in cMRI and a moderate hypometabolism of the cerebellar hemispheres in the FDG-PET (Endres et al., 2015). Based on such cases, one might speculate that neuronal dysfunction, possibly via cytotoxic T-cell activity, might be a different pathogenetic mechanism leading to neuropsychiatric symptoms in a subgroup of psychotic patients (Kayser et al., 2010). Alternatively, in this constellation, the intracellular antibodies may indicate the presence of a cancer that is not yet detectable with standard diagnostic means. In this constellation, the psychotic syndrome would function like an early heralding paraneoplastic syndrome and might be beneficial in that it could facilitate the early detection of a potentially treatable oncological condition.

### Steroid responsive encephalopathy associated with autoimmune thyroiditis

In patients with psychotic symptoms and cognitive impairment in the context of autoimmune thyroiditis, a steroid responsive encephalopathy with autoimmune thyroiditis (SREAT) should be considered. For screening, thyroid hormones and particularly thyroid peroxidase and thyroglobulin antibodies should be investigated (Castillo et al., 2006). In our study, thyroid antibodies were elevated in 24.7%, which is remarkably more than the estimated prevalence in the general population (Hollowell et al., 2002). In earlier studies, EEG abnormalities were found in nearly 95% of SREAT patients (most often generalized slowing). cMRI and CSF diagnostics also added to diagnostic certainty. In any case, steroid responsivity is the essential feature for diagnostic assignment (Castillo et al., 2006). Therefore, it is important to offer steroid treatment in suspect cases, since the therapeutic success even in long term and classical psychiatric presentations can be very remarkable.

### Psychotic syndromes—an immunological encephalopathy?

The issue of limbic encephalitis and related immunological encephalopathies as a possible cause for schizophreniform or also affective syndromes is still new in psychiatry. Some authors summarize all antibody associated autoimmune encephalopathies within the umbrella concept of immunological encephalopathies and propose a standardized symptomatic immunomodulatory therapeutic approach for all of these diseases including anti-VGKC- and anti-NMDAR-encephalitis as well as SREAT (Friese and Magnus, 2012). Our data illustrate that systematic diagnostic screening in fact result in high rates of diverse abnormal immunological signals. However, the therapeutic implications are still poorly understood.

### Implications for clinical diagnostic assessment and therapy

The issue of adapting clinical diagnostic and therapeutic standards to the advent of concepts of immunological encephalopathy and limbic encephalitis will most likely be very controversial. In many—if not in the majority—of psychiatric clinics, there won't be diagnostic competence for doing routine CSF studies. Additionally, many psychotic patients will not comply with doing CSF studies and in this constellation, currently, we don't do such investigations neither. The question of cost-effectiveness also arises. Does it make sense to diagnose immunological encephalopathy in a patient with psychological symptoms when it is unclear how this patient should then be treated? While such considerations question the rational of offering CSF studies to all patients with acute psychosis, there are also many arguments in favor of such a procedure. The vast majority of our psychotic patients did comply with these investigations and in fact were actively pursuing this road. One likely reason is that the disease model of “psychosis as an inflammatory brain process” was easier to accept for these patients and their families than the concept of schizophrenia. In quite a few of our immunological encephalopathy patients, the response to immune therapy was very good and in fact much better than the response to the classical variant of symptomatic psychopharmacology. These patients were happy to receive a more causal therapy than, for example, only taking symptomatic antipsychotic drugs.

## Conclusion

Clinicians must balance the potential benefits of a diagnostic procedure against its medical risks and costs. With the medical risk and financial costs of doing a CSF study being very low, and based on our data one has to judge whether it is worthwhile doing CSF studies in 100 patients in order to detect three with increased WBC counts, about 40 with some evidence of BBB disturbance, seven with OCBs, and seven with autoantibodies against some neuronal cell surface or intracellular antibodies. Given the potentially devastating course of schizophreniform syndromes, the authors of this paper feel that our data strongly point in favor of more comprehensive CSF investigations as routine procedures in psychotic patients. There is still a very large demand for research not only in the diagnostic but also in the linked therapeutic dimension. In case CSF findings like ours were clearly linked to beneficial responses to immunomodulatory treatments, this obviously would change the diagnostic and therapeutic reality in psychiatry in a dramatic way.

### Conflict of interest statement

Annette Baumgartner: Consulting and lecture fees, grant and research support from Bayer Vital GmbH, Biogen Idec, Merck Serono, Novartis, Sanofi-Aventis and Teva. Oliver Stich: Consulting and lecture fees, grant and research support from Bayer Vital GmbH, Biogen Idec, Genzyme, Merck Serono, Novartis, Sanofi-Aventis and Teva. Ludger Tebartz van Elst: Advisory boards, lectures, or travel grants within the last three years: Eli Lilly, Janssen-Cilag, Novartis, Shire, UCB, GSK, Servier, Janssen, and Cyberonics. Dominique Endres, Evgeniy Perlov, Tilman Hottenrott, and Rick Dersch declare that the research was conducted in the absence of any commercial or financial relationships that could be construed as a potential conflict of interest.
