# Supplementary material for: Immunological findings in psychotic syndromes: a tertiary care hospital's CSF sample of 180 patients
Source: Front Hum Neurosci. 2015 Sep 10;9:476. doi: 10.3389/fnhum.2015.00476 (PMC4564575; doi:10.3389/fnhum.2015.00476)
Supplement: Supplementary file 1 [file Table1.DOCX]

**Supplemental Material**

**SUPPLEMENTAL TABLE 1: CSF Diagnostics in Earlier Studies (adapted from Vasic et al., 2012).**

| Study | Collective | Cell count | BBB dysfunction | Intrathecal immunoglobulin-synthesis |
| --- | --- | --- | --- | --- |
| Bechter et al., 2010 | N=63; Treatment resistant  affective and schizophrenic spectrum disorder | Slightly increased CSF cell counts (5–8/lL) in 10% | In 29% increased albumin quotient | In 14% intrathecal humoral immune responses |
| Nikkilä et al., 2001 | N= 30; Acute psychotic patients | Increased frequency of activated lymphoid cells; decreased frequency of normal lymphocytes | - | - |
| Nikkilä et al., 1999 | N=35; Acute schizophrenia | Proportion of mononuclear phagocytes/ macrophages was initially significantly higher than that in the control group | - | - |
| Schwarz et al., 1998 | N=40; Schizophrenia | Slightly increased WBC count (5-8/ µ l) in 7.5% | In 17.5% elevated albumin quotient | In 5% increased immunoglobulin-G-Index > 0.7 |
| Müller and Ackenheil, 1995 | N=27; Schizophrenia | - | Increased albumin quotient in 22% | In 15% elevations of immunoglobulin-G |
| Kirch et al., 1992 | N=46; Schizophrenia |  | Increased albumin quotient in 22% | In 20% endogenous  central nervous system immunoglobulin-G production |
| Bauer and Kornhuber, 1987 | N=15; Schizophrenia | - | In 54% BBB-dysfunction | No endogenous central nervous system immunoglobulin-G  production |
| Kirch et al., 1985 | N=24; Schizophrenia | - | Increased albumin quotient in 29% | Elevated endogenous central nervous system immunoglobulin-G in 33% |
| Torrey et al., 1985 | N=58; Schizophrenia and schizoaffective disorder | - | Increased albumin quotient in 7% | - |
| Axelsson et al., 1982 | N= 25; Schizophrenia | - | Increased albumin quotient in 28% | - |
| Delisi et al., 1981 | N= 35; Chronic schizophrenia | - | - | Decreased immunoglobulin-G level in CSF |
| Albrecht et al., 1980 | N=60; Schizophrenia | - | No significant increased frequency of BBB dysfunction in patient group | Increased CSF/serum antibody ratio for CMV, vaccinia, HSV-1, and for influenza virus |
| Toorey et al., 1978 | N=66; Functional psychoses | - | - | Elevations of immunoglobulin-G or measles antibodies |
| Hunter et al., 1969 | N=256; General  psychiatric inpatients | - | Protein concentration increased in 14% of general psychiatric inpatients | - |
| Bruetsch, 1942 | N=634; Schizophrenia | - | Protein concentration increased in 4.4% of male and 2.7% of female patients | - |

*WBC count, white blood cell count; BBB, blood-brain-barrier; CMV, cytomegalovirus; HSV-1, herpes simplex virus type 1.*

**References**

Albrecht, P., Torrey, E. F., Boone, E., Hicks, J. T., and Daniel, N. (1980). Raised cytomegalovirus-antibody level in cerebrospinal fluid of schizophrenic patients. *Lancet* 2 (8198), 769–72.

Axelsson, R., Martensson, E., and Alling, C. (1982). Impairment of the blood-brain barrier as an aetiological factor in paranoid psychosis. *Br J Psychiatry* 141, 273–81.

Bauer, K., and Kornhuber, J. (1987). Blood-cerebrospinal fluid barrier in schizophrenic patients. *Eur Arch Psychiatry Neurol Sci* 236 (5), 257–59.

Bechter, K., Reiber, H., Herzog, S., Fuchs, D., Tumani, H., and Maxeiner, H. G. (2010). Cerebrospinal fluid analysis in affective and schizophrenic spectrum disorders: identification of subgroups with immune responses and blood-CSF barrier dysfunction. *J Psychiatr Res* 44 (5), 321-30.

Bruetsch, W.L., Bahr, M.A., Skobba, J.S., and Dieter, W.J. (1942). The group of dementia praecox patients with an increase of the protein content of the cerebrospinal fluid. *J Nerv Ment Dis* 95, 669–679.

Delisi, L. E., Weinberger, D. R., Potkin, S., Neckers, L. M., Shiling, D. J., and Wyatt, R. J. (1981). Quantitative determination of immunoglobulins in CSF and plasma of chronic schizophrenic patients. *Br J Psychiatry* 139, 513–18.

Hunter, R., Jones, M., and Malleson, A. (1969). Abnormal cerebrospinal fluid total protein and gamma-blobulin levels in 256 patients admitted to a psychiatric unit. *J. Neurol. Sci.* 9 (1), 11–38.

Kirch, D. G., Alexander, R. C., Suddath, R. L., Papadopoulos, N. M., Kaufmann, C. A., Daniel, D. G., and Wyatt, R. J. (1992). Blood-CSF barrier permeability and central nervous system immunoglobulin G in schizophrenia. *J. Neural Transm. Gen. Sect.* 89 (3), 219–32.

Kirch, D. G., Kaufmann, C. A., Papadopoulos, N. M., Martin, B., and Weinberger, D. R. (1985). Abnormal cerebrospinal fluid protein indices in schizophrenia. *Biol. Psychiatry* 20 (10), 1039–46.

Müller, N., and Ackenheil, M. (1995). Immunoglobulin and albumin content of cerebrospinal fluid in schizophrenic patients: relationship to negative symptomatology. *Schizophr. Res.* 14 (3), 223–28.

Nikkilä, H. V., Müller, K., Ahokas, A., Miettinen, K., Rimón, R., and Andersson, L. C. (1999). Accumulation of macrophages in the CSF of schizophrenic patients during acute psychotic episodes. *Am J Psychiatry* 156 (11), 1725–29.

Nikkilä, H. V., Müller, K., Ahokas, A., Rimón, R., and Andersson, L. C. (2001). Increased frequency of activated lymphocytes in the cerebrospinal fluid of patients with acute schizophrenia. *Schizophr. Res.* 49 (1-2), 99–105.

Schwarz, M. J., Ackenheil, M., Riedel, M., and Müller, N. (1998). Blood-cerebrospinal fluid barrier impairment as indicator for an immune process in schizophrenia. *Neurosci. Lett.* 253 (3), 201–03.

Toorey, E. F., Peterson, M. R., Brannon, W. L., Carpenter, W. T., Post, R. M., and van Kammen, D. P. (1978). Immunoglobulins and viral antibodies in psychiatric patients. *Br J Psychiatry* 132, 342–48.

Torrey, E. F., Albrecht, P., and Behr, D. E. (1985). Permeability of the blood-brain barrier in psychiatric patients. *Am J Psychiatry* 142 (5), 657–58.

Vasic, N., Connemann, B. J., Wolf, R. C., Tumani, H., and Brettschneider, J. (2012). Cerebrospinal fluid biomarker candidates of schizophrenia: where do we stand? *Eur Arch Psychiatry Clin Neurosci* 262 (5), 375-91.
